# Xanthogranulomatous prostatitis mimicking prostate carcinoma: a case report and comprehensive literature review

**DOI:** 10.3389/fmed.2026.1883799

**Published:** 2026-08-25

**Authors:** Mingfeng Ji, Yihao Chen, Haoxuan Feng, Jiasong Wang, Haozhen Li, Heyao Tong, Zhuwei Song, Qimin Wang, Zhihong Dai, Bo Fan

**Affiliations:** 1 Department of Urology, Second Affiliated Hospital of Dalian Medical University, Dalian, Liaoning, China; 2 Liaoning Provincial Key Laboratory of Urological Digital Precision Diagnosis and Treatment, Dalian, Liaoning, China; 3 Liaoning Engineering Research Center of Integrated Precision Diagnosis and Treatment Technology for Urological Cancer, Dalian, Liaoning, China; 4 Dalian Key Laboratory of Prostate Cancer Research, Dalian, Liaoning, China; 5 Department of Urology, Chaoyang Central Hospital, Chaoyang, Liaoning, China; 6 Department of Urology, Renhe Hospital, Shanghai, China; 7 Department of Pathology, Second Affiliated Hospital of Dalian Medical University, Dalian, Liaoning, China

**Keywords:** case report, granulomatous inflammation, prostatic carcinoma, therapy, xanthogranulomatous, xanthogranulomatous prostatitis

## Abstract

**Introduction:**

Granulomatous prostatitis (GP) is a relatively uncommon disorder, accounting for approximately 0. 8% to 1.0% of all benign inflammatory prostatic conditions. Xanthogranulomatous prostatitis (XGP) is an exceptionally rare subtype of GP, with only a limited number of cases documented in the medical literature. Clinically and radiologically, this entity often mimics prostatic carcinoma, necessitating histopathological examination for an accurate diagnosis.

**Case presentation:**

A 69-year-old man presented with a multi-year history of urinary frequency, nocturia, dysuria, and dull flank pain. Clinical evaluation and laboratory studies revealed an elevated prostate-specific antigen (PSA) level. Ultrasonography demonstrated prostatic enlargement with heterogeneous echogenicity and a 1.7-cm intravesical protrusion. Prostate magnetic resonance imaging (MRI) indicated a prostatic volume of 46.33 cm^3^. A lesion measuring up to 2.4 cm in maximum diameter was localized to the right basal transitional zone and right basal fibromuscular stroma. The lesion exhibited low signal intensity on T2-weighted imaging, hyperintensity on diffusion-weighted imaging (DWI), and restricted diffusion with a correspondingly low signal on the apparent diffusion coefficient (ADC) map. Bladder involvement was also noted, yielding a Prostate Imaging Reporting and Data System (PI-RADS) score of 4. The patient subsequently underwent transurethral resection of the prostate (TURP) as a therapeutic intervention. Histopathological examination of the resected tissue confirmed the diagnosis of XGP.

**Conclusion:**

XGP is an extremely rare prostatic pathology, and histopathological examination remains the gold standard for definitive diagnosis. Conservative management and TURP constitute the recommended therapeutic approaches for this condition.

## 1 Introduction

Granulomatous inflammation of the prostate constitutes an uncommon subtype of prostatic inflammatory conditions, characterized by its low prevalence in clinical practice. The classification scheme for granulomatous prostatitis (GP) remains controversial; however, it generally encompasses four distinct categories, infective, iatrogenic, allergic, and non-specific. Non-specific granulomatous inflammation represents the most prevalent variant, with XGP being one of its subtypes. The clinical presentation and characteristics of XGP closely resemble those of prostatic carcinoma, typically manifesting as lower urinary tract symptoms accompanied by characteristically elevated PSA levels, which may reach as high as 172.5 ng/mL (1). Imaging modalities, including transrectal ultrasound and magnetic resonance imaging (MRI), are unable to reliably differentiate between xanthogranulomatous prostatitis and primary prostatic carcinoma, frequently resulting in misdiagnosis of XGP as malignancy (1–4). Consequently, histopathological examination remains the gold standard for XGP diagnosis (5). Therapeutic approaches for XGP continue to be debated among clinicians. We report a case of xanthogranulomatous prostatitis treated in our hospital's Department of Urology and provide a comprehensive literature review summarizing the clinical features, diagnostic approaches, and therapeutic management of XGP.

## 2 Case presentations

**Figure 1:**
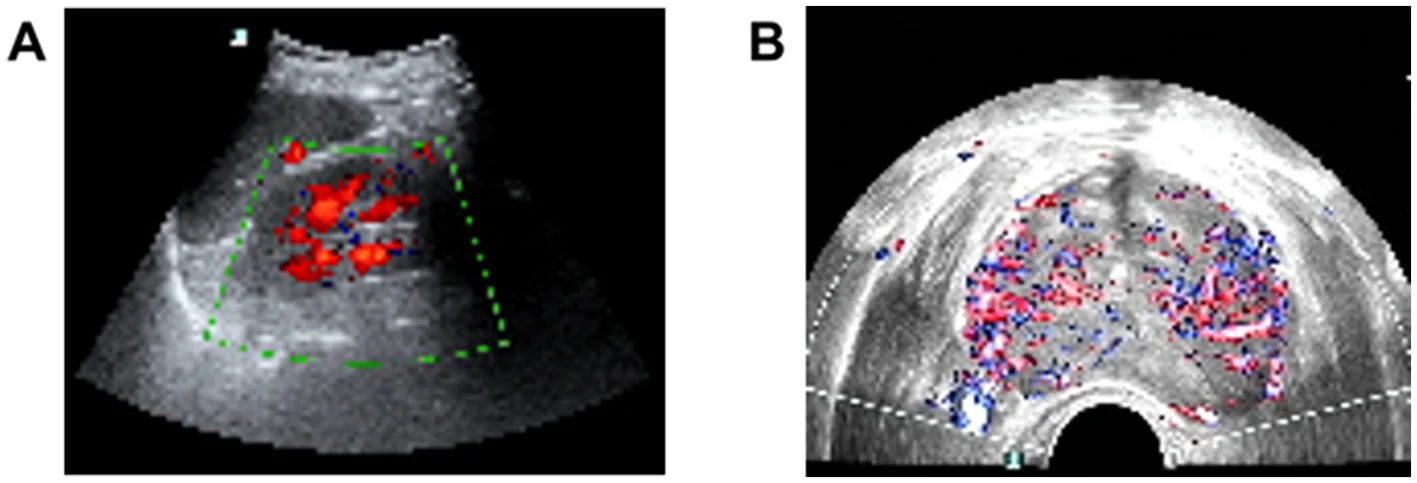
**(A)** Ultrasonography showed an enlarged prostate gland, and inhomogeneous echogenicity, **(B)** Transrectal elasticity and ultrasound of prostate.

A 69-year-old man presented with a several-year history of urinary frequency, nocturia, dysuria, and dull flank pain. Laboratory examination revealed an elevated PSA level. Ultrasonography demonstrated prostatic enlargement with 1.7 cm intravesical protrusion, inhomogeneous echogenicity, and post-void residual urine of 10 mL (Figure 1A). Digital rectal examination (DRE) revealed a firm, enlarged prostate without palpable nodules or tenderness. Laboratory values showed tPSA: 32.724 ng/mL, f/tPSA ratio: 0.03, PSAD: 0.533 ng/mL/mL, and prostate health index (PHI): 124.96. Transrectal elastography and ultrasound of the prostate revealed an intact prostatic capsule with indistinct boundaries between the transition and peripheral zones. Marked heterogeneity of prostatic tissue was observed, with patchy and mass-like hyperechoic and hypoechoic alterations. Color Doppler flow imaging (CDFI) and contrast-enhanced Doppler (CDE) demonstrated increased vascularity, predominantly in the peripheral zone of the apex and mid-gland, with symmetric distribution. Prostatic urethral length measured 4.3 cm, membranous urethral length 1.2 cm, and urethral length from the verumontanum to bulbocavernosus muscle segment 3.2 cm (Figure 1B). Strain elastography scores were: prostatic parenchyma 3–4, prostatic urethra 3, and membranous urethra 3. Shear-wave elastography values (kPa) were: prostatic parenchyma 30, prostatic urethra 10, and membranous urethra 10. Three-dimensional modeling demonstrated intact continuity of the external urethral sphincter, puborectalis muscle, internal anal sphincter, or external anal sphincter.

**Figure 2:**
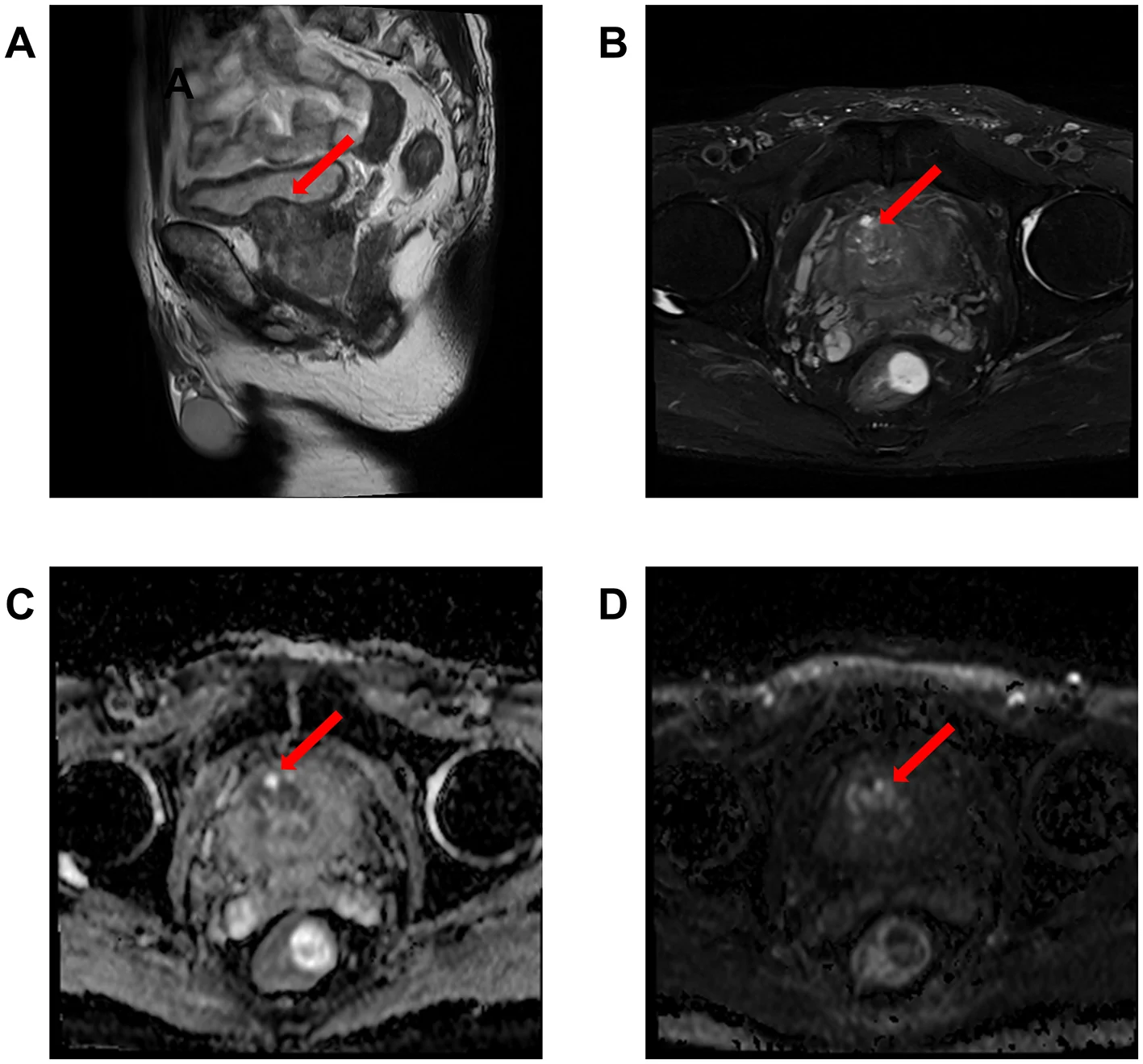
**(A)** Prostate protruding into the bladder. **(B)** Prostate MRI scan T2WI showed low signal. **(C)** Prostate MRI scan ADC showed low signal. **(D)** Prostate MRI scan DWI revealed hyperintense signal.

**Figure 3:**
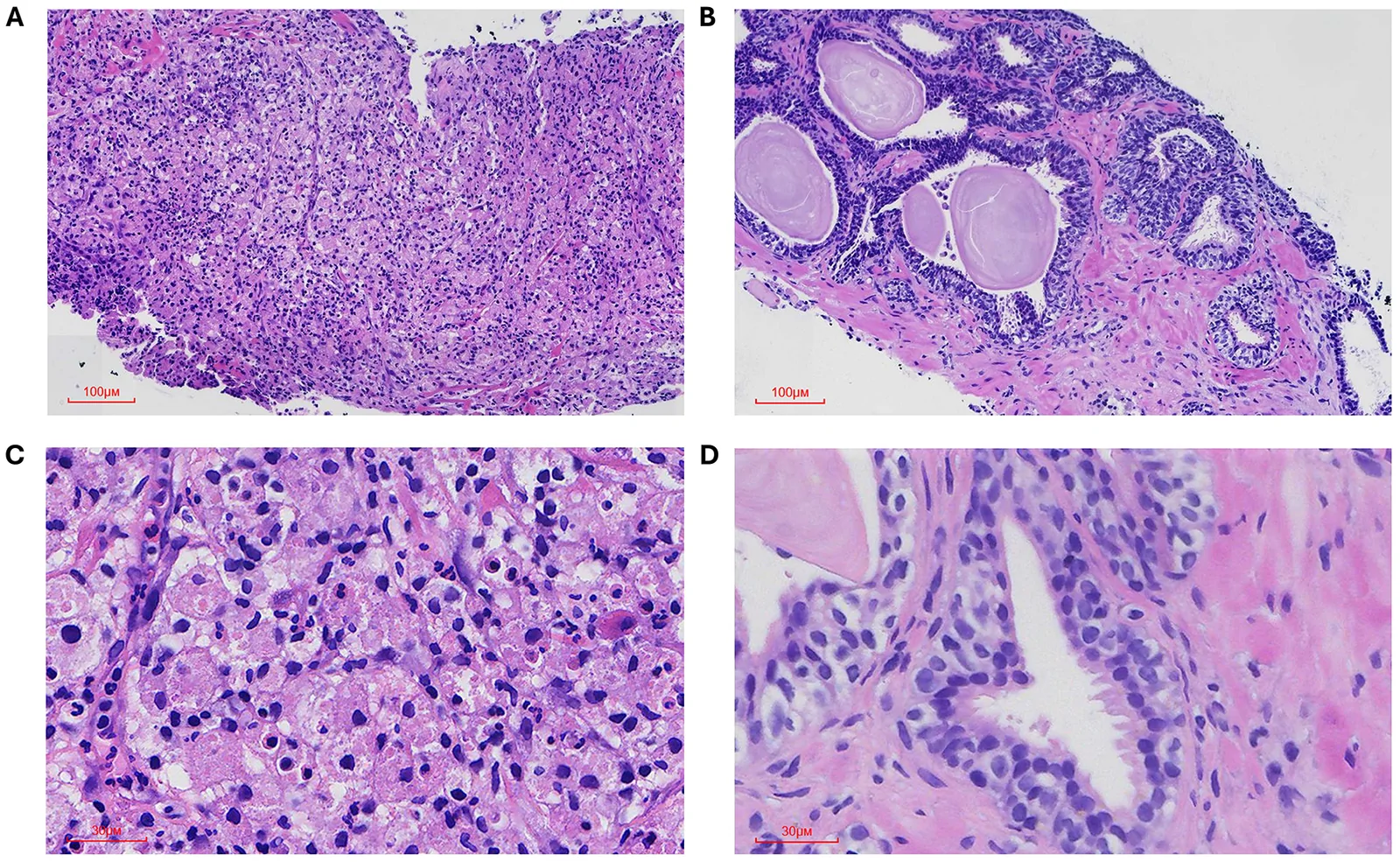
Paraffin HE section **(A)** low and **(C)** high images of XPG, foam cells and epithelioid hyperplasia were found in some areas. Foam cells gathered in some areas. There were many lymphocytes, plasma cells and neutrophils infiltration in the lesions, accompanied by necrosis. Paraffin H&E section **(B)** low and **(D)** high images of the prostatic tissue without inflammation.

**Figure 4:**
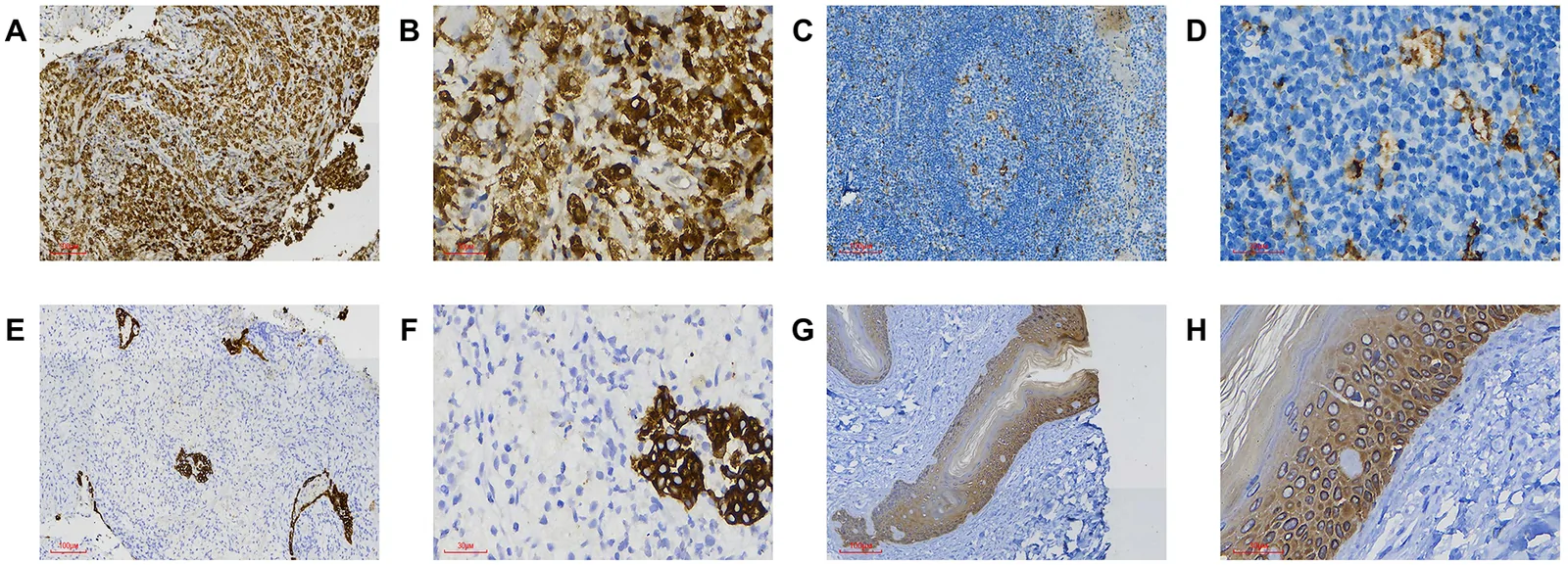
Immunohistochemical staining **(A)** low and **(B)** high on CD68+ of XPG, positive contrast immunohistochemical staining **(C)** low and **(D)** high on CD68+. Immunohistochemical staining **(E)** low and **(F)** high on CK(AE1/AE3)– of XPG, positive contrast immunohistochemical staining **(G)** low and **(H)** high onCK(AE1/AE3)+.

Prostate biparametric MRI (bpMRI) demonstrated a prostate measuring 5.3 × 4.1 × 4.1 cm. The lesion was located in the right basal transitional zone and right basal fibromuscular stroma, with a maximum diameter of 2.4 cm. T2-weighted imaging showed low signal intensity, diffusion-weighted imaging revealed hyperintense signals, and apparent diffusion coefficient mapping demonstrated low signal intensity. Bladder involvement was noted, based strictly on the bpMRI findings, the lesion was assigned a PI-RADS score of 4 (Figure 2). We obtained the pathological results by 12-core systematic transperineal with 2-core targeted biopsy prostate biopsy as follows: xanthogranulomatous prostatitis (Figures 3A, C), while corresponding prostatic tissue without inflammation is presented in Figures 2B, C for morphological comparison, with immunohistochemical staining showing CD68 (+) and cytokeratin AE1/AE3 (–) (Figures 4A, B, E, F), and corresponding CD68 (+) and AE1/AE3 (+) are presented in Figures 4C, D, G, H for morphological comparison. The International Prostate Symptom Score (IPSS) was 27, and quality of life questionnaire (QoL) score was 5. Given the presence of severe obstructive urinary symptoms, a transurethral resection of the prostate (TURP) procedure was carried out. Subsequent histopathological examination revealed the presence of xanthogranulomatous inflammation, which was characterized by the aggregation of foam cells, lymphocytes, plasma cells, and neutrophils. On postoperative day 5, the urethral catheter was removed. Subsequent observation revealed that the patient exhibited unobstructed and smooth micturition, with no evidence of urinary incontinence.

## 3 Discussion

**Figure 5:**
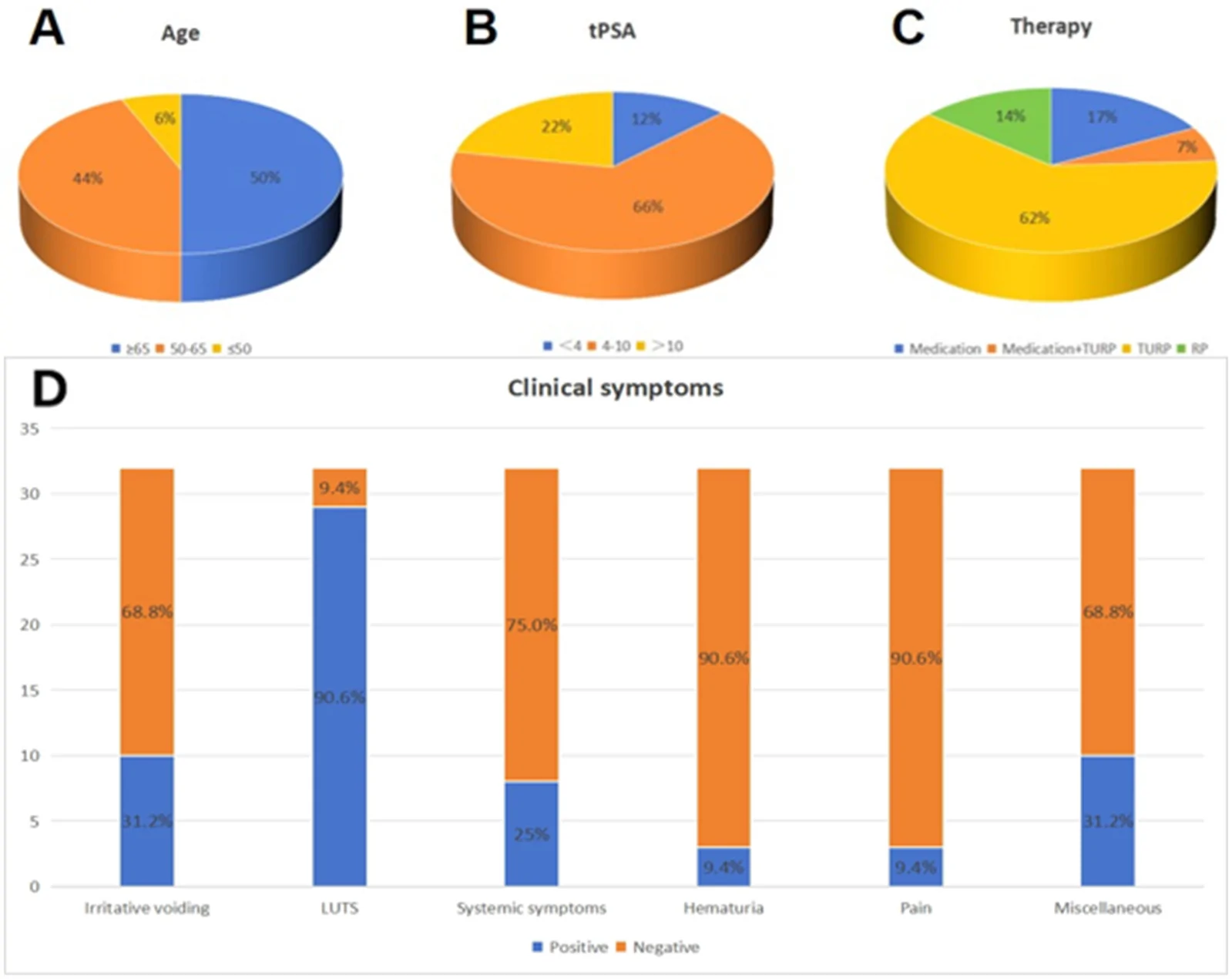
**(A)** Age composition of patients (year), **(B)** tPSA composition of patients (ng/ml), **(C)** Therapy composition of patients, **(D)** Clinical symptoms composition of patients.

XGP represents an exceedingly uncommon prostatic disorder that frequently exhibits clinical manifestations and radiological features that closely resemble those of prostatic carcinoma, with fewer than 40 cases reported to date (3, 6–8). The clinical presentation most commonly manifests as lower urinary tract symptoms, such as nocturia, dysuria, with typically elevated PSA level (1). To systematically review this rare entity, we searched the PubMed and Embase using the keywords “Xanthogranulomatous prostatitis,” after screening titles and abstracts, we identified 20 articles published between 1986 and 2024, encompassing 32 patients (Table 1). Among the 32 patients, the age at diagnosis ranged from 46 to 81 years, with a mean age of 64.2 years, half (50%) of the patients are ≥65 years old (Figure 5A). The majority (88%) of patients with elevated PSA, of which 66 % of patients with PSA > 10 ng/ml (Figure 5B).

**Table 1:** Literatures of xanthogranulomatous prostatitis.

Clinical symptoms								DRE									MRI features						
Study	Age	Irritative voiding	LUTS	Systemic symptoms	Hematuria	Pain	Miscellaneous	tPSA	Prostate size (ml)	Ultrasound features	Enlarged	Hard	Tender	Nodule	Routine urinalysis	Urine culture	T1	T2	DWI	ADC	Immunohistochemistry	Therapy	Follow-up
Matsumoto et al. (28)	62		√	√				NA	NA	Hypoechoic				√	NA	NA	NA				NA	NA	NA
Miekoś et al. (25)	58		√	√				NA	NA	NA					Leukocytosis	*Proteus vulgaris*	NA				NA	RP	6-month: intractable urinary tract infection and a stone in the post adenomectomy bed
	46		√			√	Epididymitis	NA	NA	NA	√		√	√	Leukocytosis	*E. coli*	NA				NA	TURP	3-month: urine retention was less than 20 ml, urinalysis was normal
Mohan et al. (29)	67		√					2.8	NA	NA		√		√	NA	NA	NA				NA	TURP	NA
	70	√						10	NA	NA		√		√	NA	NA	NA				NA	NA	NA
Rafique and Yaqoob (30)	60		√					150	50	Hypoechoic	√	√		√	Leukocytosis	(–)	NA				NA	TURP	16-month:symptom free, PSA has decreased to 6 ng/ml
Valsangkar et al. (31)	52		√	√				NA	52	Hypoechoic	grade II				NA	NA	NA				NA	antibiotic +TURP	1- week: mild urge incontinence was settled
Lee et al. (10)	74		√				Epididymo-orchitis	11.74	74	Hypoechoic	√			√	NL	(–)	Hypointense	Hyperintense	NA	Hypointense	CD 68(+); PSA(-); cytokeratin(-)	alpha-blocker	NA
Pastore et al. (32)	51–62	√(5/5)	√(5/5)	√(3/5)			Hematospermiai (5/5)	4.8–6.7	NA	Hypoechoic	√	√		√	NA	NA	NA				NA	TURP	5-week: normal IPSS scores and normal uroflowmetry
Cheng et al. (6)	60		√					6.5	NA	NA	grade II	√			NA	NA	Iso-intensity	Hyperintense	Hyperintense	Hypointense	CD 68(+); PSA(-)	NA	NA
	71		√		√			5.5	NA	NA	grade III				NA	NA	Iso-intensity	Hyperintense	Hyperintense	Hypointense	NA	antibiotic	1-month: PSA decreased to 2.4 ng/ml
Wollin et al. (33)	78		√					41.4	328.4	NA	√	√			NA	NA	Gland was very heterogeneous and lobular				NA	RP	voiding well, low post-void residual
Kumbar et al. (8)	67	√						6	NA	NA		√		√	NA	NA	NA				NA	TURP	NA
	63	√	√					5.5	NA	NA		√		√	NA	NA	NA				NA	TURP	NA
	81	√	√					4.8	NA	NA		√		√	NA	NA	NA				NA	TURP	NA
	65	√	√					5.2	NA	NA		√		√	NA	NA	NA				NA	TURP	NA
	75		√					6.2	NA	NA		√		√	NA	NA	NA				NA	TURP	NA
Xing et al. (1)	75		√				Rectal bleeding	172.5	42.5	Inhomogen- -eous echogenicity		√		√	NA	NA	NA	Prostate-rectal fistula	NA	NA	CD 68(+); PSA(–)	Tamsulosin + estrogen	4-month: symptom free and PSA began to decrease;17 months: PSA decreased to < 4 ng/mL
Noyola et al. (34)	47		√					0.94	NA	NA	√	√			NA	NA	NA				NA	TURP+RP	NA
Shukla et al. (7)	65		√					8.85	52	NA		√		√	NL	NA	NA				NA	TURP	NA
	69		√					NA	78	NA		√		√	NL	NA	NA				NA	TURP	NA
Jabbour et al. (35)	59		√	√	√			0.54	106.32	NA	√	√	√		Leukocytosis	(–)	NA				NA	TURP	NA
Belga et al. (36)	70			√	√	√		6	NA	NA	√		√	√	NA	*Pseudomo- -nas aeruginosa*	NA				AE 1/AE 3(–)	TURP + antibiotic	3-month: no infectious or urinary tract symptoms
Joseph (24)	65		√			√	Tenesmus	0.43	33	NA	NL				NA	NA	NL				NA	Tamsulosin + Cymbalta + antibiotic	1-year: PSA PSA level was 1.26 ng/mL
Al-Naimi et al. (26)	63		√				Epididymo-orchitis	6	29	NA	√				NA	*E. coli*	NA				NA	TURP	NA
Tang et al. (9)	56		√					49.19	NA	Hypoechoic	√				NL	NA	NA	Hypointense	Hyperintense	Hypointense	NA	antibiotic	2-month: dysuria symptom had disappeared
Okamoto et al. (3)	77	No symptoms						10.4	NA	NA	√	√			NL	(–)	NA	Moderate signal	NA	NA	NA	antibiotic + open suprapubic tumor resection	15-month: symptom-free
Hassan et al. (2)	75		√					90	70	Hypoechoic	√	√		√	NA	NA	NA				LCA(+); CD 68(+)	TURP	12-month: symptom-free, PSA has decreased to 4.5 ng/ml

Lower urinary tract symptoms were present in most patients (90.6%), with some patients experiencing irritative voiding symptoms, systemic symptoms, hematuria and pain (Figure 5D). The present case report demonstrated a typical presentation of xanthogranulomatous prostatitis, with LUTS (frequency, nocturia, dysuria) present for at least 1 year. Digital rectal examination reveals an enlarged, hard prostate with or without nodules and tenderness in most patients. Although multiparametric MRI and PET-CT can be helpful in the diagnosis of XGP, they also have limitations. MRI may show false-positive results in inflammatory conditions, making XGP look like prostate cancer. Previous studies have found discordant results between mpMRI and PSMA-PET. For example, Tang et al. reported an XGP case with a false-positive mpMRI but a true-negative ^68^Ga-PSMA PET/CT (9). This discordance suggests that PSMA-PET may help avoid misdiagnosis when MRI results are unclear. In the present case, although the patient had an elevated PSA level, PSMA-PET was not performed prior to surgery due to financial considerations and the high cost of the examination, and the patient underwent a whole body bone scan, the results showed that no malignant tumor lesions were found. Therefore, definitive diagnosis still relies on the histopathological examination of prostate biopsy specimens, which typically demonstrates xanthogranulomatous inflammation composed of foamy histiocytes, plasma cells, lymphocytes, and neutrophils (9, 10).

The precise etiology of xanthogranulomatous inflammation remains elusive. It has been documented to have an association with hyperlipidemia (11). Mbakop A proposed that it frequently results from epithelial destruction of the epithelium and extravasation of prostatic secretions in the stroma and Tanner also advanced that occlusion of ducts evacuating glandular secretion seems to play an important part in the pathogenesis of XGP (12, 13). The consequent epithelial disruption results in the efflux of cellular debris, bacterial toxins, and prostatic secretions, including corpora amylacea, into the stroma. This process subsequently elicits an intense localized inflammatory response (14). Alexander RB proposed that it may be an autoimmune disorder characterized by an HLA-DR15-linked T-cell response against proteins in prostatic secretions (15).

Developed in the 1990s, elastography provides a non-invasive and non-destructive assessment of the mechanical properties of soft tissues (16, 17). Currently, it is widely used to differentiate benign from malignant thyroid and breast tumors (18–20), and its clinical application has been extended to the evaluation of prostate lesions. The underlying mechanism in prostate cancer involves increased microvessel density and collagen deposition surrounding the lesions, which results in greater tissue stiffness (21). Previous studies have reported that a strain elastography (SE) score > 3 is the optimal cut-off value for detecting malignancy (22). Additionally, Tyloch et al. demonstrated that for shear-wave elastography (SWE), the optimal cut-off values for diagnosing prostate cancer are 45 kPa in the transition zone and 35 kPa in the peripheral zone (23). In the present case, the patient exhibited an SE score of 3–4, which falsely indicated a high risk of malignancy. This false-positive result was likely attributable to severe focal fibrosis caused by XGP, which increased the stiffness of the lesion. However, the absolute SWE value was only 30 kPa. Given that the lesion was located in the right basal transition zone, this value was well below the 45 kPa threshold for malignancy in this specific region (23). These findings suggest that although XGP can mimic prostate cancer on SE, quantitative SWE may assist clinicians in differentiating it from true malignancy.

In the treatment of XGP, conservative management is recommended for patients with mild or tolerable symptoms, including the use of alpha-blockers and antimicrobial agents (24, 25). In cases where the patient present with severe obstructive urinary symptoms, surgical intervention in the form of TURP or prostatectomy may be indicated and subsequently carried out (26). This approach is consistent with findings from previous case reports (Figure 5C). In the present case, given the patient's several-year history of urinary frequency, nocturia, dysuria, and dull flank pain, TURP was performed. During follow-up, the majority of the patient's symptoms showed significant improvement. Research findings have indicated that 19% of patients diagnosed with non-specific granulomatous prostatitis subsequently develop prostate cancer (PCa). Consequently, individuals with persistently elevated serum prostate–specific antigen (PSA) levels require close and regular surveillance to facilitate early detection and intervention (27).

A limitation in the diagnostic evaluation of this patient was the inability to perform multiparametric magnetic resonance imaging (mpMRI). Dynamic contrast-enhanced (DCE) MRI was omitted due to the patient's allergy to gadolinium-based contrast agents. Consequently, the patient underwent bpMRI, consisting of T2-weighted and diffusion-weighted imaging (DWI/ADC) without intravenous contrast administration. In the absence of DCE imaging, the reported PI-RADS score of 4 was based strictly on the bpMRI findings.

## 4 Conclusion

XGP represents an extremely uncommon prostate-related disease, with histopathological diagnosis commonly regarded as the gold standard. The clinical symptoms of most patients are LUTS and elevated PSA. Depending on individual patient conditions, conservative management and TURP are recommended for XGP. Close attention should be paid to symptom improvement and PSA levels during follow-up monitoring.

## Data Availability

The raw data supporting the conclusions of this article will be made available by the authors, without undue reservation.
